# Association Study between Polycystic Ovarian Syndrome and the Susceptibility Genes Polymorphisms in Hui Chinese Women

**DOI:** 10.1371/journal.pone.0126505

**Published:** 2015-05-15

**Authors:** Lingxia Ha, Yuhua Shi, Junli Zhao, Tao Li, Zi-Jiang Chen

**Affiliations:** 1 Center for Reproductive Medicine, Provincial Hospital Affiliated to Shandong University, National Research Center for Assisted Reproductive Technology and Reproductive Genetics, The Key laboratory for Reproductive Endocrinology of Ministry of Education, Shandong Provincial Key Laboratory of Reproductive Medicine, Reproductive Medical Hospital Affiliated to Shandong University, Jinan, 250021, China; 2 Center for Reproductive Medicine, General Hospital of Ningxia Medical University, Yinchuan, 75004, China; 3 Renji Hospital, Shanghai Jiao Tong University School of Medicine, Shanghai, 200000, China; Peiking University Third Hospital, CHINA

## Abstract

**Introduction:**

Polycystic ovary syndrome (PCOS) is one of the most common endocrine-metabolic disorders. Evidence of familial aggregation analysis and different clinical traits among different regions and ethnicities indicated that the pathogenesis of PCOS is associated with multiple genetic and environmental factors. Our previous research had identified three susceptibility loci (rs2479106, DENND1A; rs13405728, LHCGR; rs13429458, THADA) for PCOS in Han Chinese women. The overall aim of this study was to investigate the relationship between three susceptibility gene polymorphisms and PCOS in Hui ethnic women.

**Methods:**

151 patients with PCOS (case group) and 99 healthy women (control group) were recruited from the Reproductive Medicine Center of the General Hospital of Ningxia Medical University. Clinical data and serum hormone characteristics of case and control groups were collected and analyzed. The three susceptibility single-nucleotide polymorphisms have been replicated in both case and control groups. Gene polymorphisms were detected by direct sequencing after polymerase chain reaction.

**Results:**

The Body Mass Index, LH, LH/FSH ratio and total testosterone were significantly elevated in PCOS patients compared to control group (P<0.05). The frequencies of genotype and allele in rs13405728 were significantly different between the PCOS and the control groups (P<0.05). Of the SNP rs13405728, the PCOS cases with TT genotype stayed at a higher level of total testosterone, TG and LDL than those with the CC and CT genotypes. In contrary, there was no statistical difference between the two groups for SNP rs13429458 and rs2479106 (P>0.05).

**Conclusion:**

The present study suggested that the SNP rs13405728 in the LHCGR gene was associated with PCOS in Hui ethnic women, and its TT genotype characterized with higher level of TT, TG and LDL.

## Introduction

Polycystic ovary syndrome (PCOS) is one of the most common endocrine-metabolic disorders, affecting 6–8% reproductive-aged Asian women [1]^.^ It is characterized by oligomenorrhea or amenorrhea, hyperandrogenism, and polycystic ovaries [2]. Some common features like menstrual irregularity, subfertility, obesity, hirsutism, acne, and abnormal biochemistry are accompanied with raised serum testosterone, androstenedione, insulin, and luteinizing hormone level [3]. In recent times, it has also been considered as an important cardiovascular risk factor [4–5]. Women with PCOS are more likely to have poor vascular compliance, vascular endothelial dysfunction, hypertension and dyslipidemia in comparison with women without PCOS [6–9]. The disorder has detrimental impact on women's health; however, its etiologies have been poorly understood.

Evidence of familial aggregation analysis and different clinical traits among different regions and ethnicities indicated that the pathogenesis of PCOS is associated with multiple genetic and environmental factors [10]. Recently, many studies suggested that genetic factors were strongly associated with the etiology of PCOS [11–12]. However, only a few genes were found have association with PCOS or some clinical traits related to PCOS. Our previous research, using the genome-wide association study (GWAS) on PCOS in Han Chinese women, had identified three susceptibility loci for PCOS, at 2p16.3, 2p21, and 9q33.3 [13], which were also partially confirmed in studies of European cohorts [14–18]. In this further study, we were focusing on the genetic difference in Hui ethnic women with PCOS.

Ningxia is a Hui Autonomous Region in China, and about one third of the people are from the Muslim ethnic enclaves. Their beliefs, diet and lifestyle characteristics make them relatively isolated communities. Moreover, their physical characteristics, lifestyle habits and prohibition of inter-ethnic marriages made them genetically different from other populations. The prevalence of this syndrome in Hui ethnic women is unknown, and its clinical and biochemical characteristics have not yet been reported. The current study assessed the clinical, biochemical and hormonal parameters in Hui ethnic women with PCOS. Previous studies on the Ningxia Hui population indicated that the distribution of (GGN)n repeats polymorphisms of androgen receptor (AR) gene was significantly different between Ningxia Hui and other ethnic population [19]. It is important to conduct a replication study to confirm the relationship between susceptibility of three single-nucleotide polymorphisms (SNPs) rs2479106 (DENND1A), rs13405728 (LHCGR), rs13429458 (THADA) and PCOS in Ningxia Hui ethnic women.

## Subjects and Methods

### Subjects

250 women were recruited from the Reproductive Medicine Center of the General Hospital of Ningxia Medical University during September 2009 to May 2013. Of them, 151 were diagnosed as PCOS according to the Rotterdam criteria [20]. (oligo- and/or anovulation; clinical and/or biochemical signs of hyperandrogenism; and polycystic ovaries with exclusion of other causes of hyperandrogenism, such as hyperprolaetinemia, androgen-secreting tumours, Cushing’s syndrome, and nonclassical congenital adrenal hyperplasia). The diagnosis of PCOS was satisfied when two or more of the three criteria were met. Ninety-nine age-matched healthy women volunteers with regular menstrual cycles (26–34 days) and normal ovarian morphology were included as controls, and total testosterone and mFG score were evaluated for exclusion of hyperandrogenism. None of the participants had hormonal therapy for at least 3 months before the study. All subjects were unrelated Hui ethnic women, who were residents of Ningxia Hui Autonomous Region for at least 3 generations. The study was approved by the Ethics Committee of both Shandong provincial hospital of Shandong University and General Hospital of Ningxia Medical University. All subjects had been given written informed consent, and all the participants provided their written informed consent to participate in this study.

### Clinical and biochemical measurements

Weight and height were measured by standard protocol and calibrated instruments. Body mass index (BMI) was calculated as weight (kg) divided by height squared (m^2^).

Peripheral blood samples of all subjects were collected during early follicular phase (between 3–5 of the menstrual cycle) after an overnight fast. Each subject underwent a 75-g oral glucose tolerance test (OGTT). Plasma glucose levels at 0 min and 2h after OGTT were measured using oxidase method, while insulin levels were measured by enzymatic and chemiluminescent method. The homeostasis model assessment of insulin resistance (HOMA-IR) was calculated according to the following equation: fasting plasma glucose (FPG, mmol/l) × fasting insulin (FINS, mIU/l)/22.5 [21]. Follicle stimulating hormone (FSH), luteinizing hormone (LH), prolactin (PRL), total testosterone (T) and estradiol (E_2_) were measured using chemiluminescent analyzer (Beckman Coulter Inc, Fullerton, CA, USA). Total cholesterol (TC), triglycerides (TG), low-density lipoprotein (LDL) and high-density lipoprotein (HDL) were measured on an automated biochemistry analyzer (Olympus 600, Clinical Chemistry Analyser; Olympus Diagnostica Gmbh, Ireland). Blood samples, which were collected with EDTA as an anticoagulant and stored at -80°C, were prepared for genomic DNA extraction.

### Genotyping

Total genomic DNA from peripheral blood leukocytes was extracted using the blood DNA isolation kit of TianGen Company. Sequence amplification was performed by polymerase chain reaction (PCR). The primers for PCR were listed in Table 1. All cycling conditions were as follows: an initial denaturation at 95°C for 5 min, followed by 35 cycles at 95°C for 30 sec, 58°C for 30 sec and 72°C for 45 sec, with a final extension step of 7 min at 72°C in the last cycle. The fluorescence-melting curve was applied immediately to analyze following amplification, and the direct sequencing technology was used for the genotyping.

**Table 1:** Primers of the genes for PCR.

SNPs	Sequence of the primers	Annealing°C	Size of the PCR products, bp
rs13405728	F: 5’GTGGTTCTTACTCTAGCACAATGAT3’	58	341
	R: 5’CCATCCACATACTCACTTCAATATC3’	58	341
rs13479428	F: 5’CAGCGGTATGATTTCGTAGTG3’	58	560
	R: 5’GCTAAAATCTCATCACCTGGAC3’	58	560
rs2479106	F: 5’GAGCAGCCACTCAAGAAACAG3’	58	429
	R: 5’AAGCCACCATCCAGTCTCAC3’	58	429

### Statistical Analysis

The independent segregation of alleles was tested for the Hardy–Weinberg equilibrium (H-WE). Haploview 4.2 analyzed linkage disequilibrium between the three SNPs. The results of the serum hormone characteristics were expressed as the mean ± SD. Categorical data were expressed as frequencies or percentages. The differences between PCOS patients and controls in clinical and biochemical variables were evaluated by an independent sample *t*-test. Statistical analysis of allele and genotype frequencies between women with PCOS and controls was processed using the Chi-square test. Data have been presented as odds ratio (OR) and 95% confidence interval. Statistic significant level was defined as *P*<0.05. All data were analyzed by SPSS 18.0 statistical software (SPSS, Chicago, USA).

## Results

The clinical and biochemical characteristics of women with PCOS and controls were shown in Table 2. The T level, LH level, the ratio of LH/FSH and BMI value in the PCOS group were significant higher than that in the control group (all *P* <0.01).

**Table 2:** Clinical characteristics of PCOS and control group.

Characteristics	PCOS	Control	*t*-value	*P*-value
n	151	99		
Age (years)	25.65±4.54	26.00±5.16	1.82	0.17
BMI (kg/m^2^)	23.05±3.88	21.63±3.58	2.42	0.02*
FSH (mIU/mL)	5.84±1.57	5.85±1.76	0.02	0.93
LH (mIU/mL)	12.47±4.59	3.97±1.89	8.49	0.00**
LH/FSH	2.23±0.85	0.71±0.37	4.51	0.00**
T (ng/dL)	65.34±19.78	33.64±15.18	31.69	0.00**

All the women studied were during early follicular phase (between 3–5 days of the menstrual cycle). All data were expressed as mean±SD. Abbreviations: PCOS, polycystic ovary syndrome; BMI, body mass index; FSH, follicle-stimulating hormone; LH, luteinizing hormone; T, testosterone.

*. *P* < 0.05

**. *P* < 0.01

The distribution of the genotypes and allelic frequencies of the three SNPs (rs13405728, rs13429458 and rs2479106) were all listed in Tables 3 and 4. No significant deviation of genotype and allele frequencies as the H-WE was detected between the PCOS and the control groups (*P*>0.05).

**Table 3:** Genotypes and alleles of rs13405728, rs13429458 and rs2479106 in PCOS and control group.

SNP	genotype	PCOS n(%)	Control n(%)	χ^2^	*P-value*	OR (95%CI)
rs13405728	CC	11 (7.3)	12 (12.1)	6.515	0.038*	
	CT	41 (27.2)	38 (38.4)	6.515	0.038*	
	TT	99 (65.5)	49 (49.5)	6.515	0.038*	
	allele					
	C	63 (20.9)	62 (31.3)	6.968	0.008*	0.578 [0.384~0.870]
	T	239 (79.1)	136 (68.7)	6.968	0.008*	1.729 [1.149~2.603]
rs13429458	AA	107 (70.9)	72 (72.7)	2.452	0.294	
	CA	40 (26.5)	21 (21.2)	2.452	0.294	
	CC	4 (2.6)	6 (6.1)	2.452	0.294	
	allele					
	A	254 (84.1)	165 (83.3)	0.053	0.819	1.058 [0.652~1.718]
	C	48 (15.9)	33 (16.7)	0.053	0.819	0.945 [0.582~1.534]
rs2479106	AA	90 (59.6)	68 (68.7)	2.374	0.305	
	AG	50 (33.1)	24 (24.2)	2.374	0.305	
	GG	11 (7.3)	7 (7.1)	2.374	0.305	
	allele					
	A	230 (78.1)	160 (79.8)	1.506	0.220	1.318 [0.847~2.050]
	G	72 (21.9)	38 (20.2)	1.506	0.220	0.759 [0.488~1.180]

Abbreviations: PCOS, polycystic ovary syndrome; SNP, single-nucleotide polymorphisms; A, adenine; G, guanine; C, cytosine; T, thymine; OR, odds ratio; CI, confidence interval

* *P* < 0.05

**Table 4:** Clinical and metabolic characteristics of rs13405728 genotypes.

genotypes	TT	CT+CC	*t*-value	*P*-value
*n*	96	55		
Age (years)	26.740 ± 4.374	26.714 ± 4.924	0.027	0.978
BMI(kg/m^2^)	23.486 ± 3.871	23.521 ± 3.928	-0.053	0.958
FSH (IU/L)	5.721 ± 1.527	5.919 ± 1.324	-0.792	0.430
LH (IU/L)	12.390 ± 5.456	12.368 ± 4.103	0.025	0.980
LH/FSH	2.263 ± 0.976	2.201 ± 0.886	0.380	0.704
T(ng/dL)	69.470 ± 20.313	62.215 ± 21.660	2.371	0.014*
Fasting glucose (mmol/L)	4.851 ± 0.743	4.853 ± 0.610	-0.016	0.988
2h- glucose (mmol/L)	6.680 ± 4.147	6.501 ± 2.350	-0.288	0.774
Fasting insulin (mIU/L)	15.764 ± 13.160	15.999 ± 11.401	-0.109	0.913
2h- insulin (mIU/L)	84.949 ± 82.467	79.701 ± 61.115	0.405	0.686
HOMA-IR	3.367 ± 2.925	3.654 ± 2.657	-0.591	0.556
TC (mmol/L)	4.423 ± 1.064	4.291 ± 0.878	0.766	0.445
TG (mmol/L)	1.460 ± 0.706	1.381 ± 0.744	2.152	0.038*
HDL (mmol/L)	1.339 ± 0.465	1.278 ± 0.337	0.843	0.400
LDL (mmol/L)	2.707 ± 2.069	2.379 ± 0.720	2.290	0.023*

Abbreviations: BMI, body mass index; FSH, follicle-stimulating hormone; LH, luteinizing hormone; T, testosterone; HOMA-IR, the homeostasis model assessment of insulin resistance; TC, total cholesterol; TG, triglycerides; LDL, low-density lipoprotein; HDL, high density lipoprotein.

*. *P* < 0.05

The minor allele frequencies (MAF) of rs13405728 were different in the two groups, even after BMI adjustment (P = 0.008, OR = 0.578, Padjusted = 0.012). There was no statistical difference for rs13429458 and rs2479106.

In SNP rs13405728, the frequencies of genotypes CC、CT and TT in control group were 12.1%(12 of 99), 38.4%(38 of 99) and 49.5%(49 of 99), respectively; whereas they were 7.3%(11 of 151), 27.2% (41 of 151) and 65.5%(99 of 151) in the patients with PCOS. The C allele frequency of PCOS was 20.9%, which was lower than the controls (*P*<0.01). No significant differences were found in the frequencies of genotypes and alleles of the SNPs rs13429458 and rs2479106 between PCOS and the controls (*P* >0.05).

Of the SNP rs13405728, the PCOS cases with TT genotype displayed higher level of testosterone, TG and LDL than those with the CC and CT genotypes (all *P* < 0.05; Table 4).

In both SNPs (rs13429458 and rs2479106), we have not found any significant differences in all the clinical and metabolic characteristics in different genotypes of PCOS patients (*P* > 0.05, Tables 5 and 6).

**Table 5:** Clinical and metabolic characteristics of rs13429458 genotypes.

genotypes	AA	CA+CC	*t*-value	*P*-value
*n*	107	44		
Age (years)	24.495 ± 3.661	24.535 ± 3.936	-0.059	0.953
BMI(kg/m^2^)	23.328 ± 3.902	23.901 ± 3.783	-0.827	0.410
FSH (IU/L)	5.846 ± 1.443	5.648 ± 1.452	0.766	0.445
LH (IU/L)	13.471 ± 11.455	11.919 ± 5.024	0.864	0.389
LH/FSH	2.244 ± 0.931	2.187 ± 0.984	0.337	0.737
T(ng/dL)	67.044 ± 21.423	66.696 ± 21.610	0.091	0.928
Fasting glucose (mmol/L)	4.865 ± 0.771	4.820 ± 0.481	0.358	0.721
2h- glucose (mmol/L)	6.562 ± 4.107	6.740 ± 1.972	-0.273	0.785
Fasting insulin (mIU/L)	16.279 ± 13.499	14.830 ± 9.890	0.643	0.521
2h- insulin (mIU/L)	85.242 ± 80.253	77.935 ± 62.770	0.538	0.591
HOMA-IR	3.604 ± 3.071	3.151 ± 2.137	0.891	0.374
TC (mmol/L)	4.373 ± 0.960	4.377 ± 1.089	-0.109	0.985
TG (mmol/L)	1.413 ± 0.698	1.531 ± 0.829	-0.897	0.371
HDL (mmol/L)	1.334 ± 0.398	1.280 ± 0.471	0.721	0.472
LDL (mmol/L)	2.629 ± 1.943	2.455 ± 0.894	0.569	0.570

Abbreviations: BMI, body mass index; FSH, follicle-stimulating hormone; LH, luteinizing hormone; T, testosterone; HOMA-IR, the homeostasis model assessment of insulin resistance; TC, total cholesterol; TG, triglycerides; LDL, low-density lipoprotein; HDL, high density lipoprotein.

**Table 6:** Clinical and metabolic characteristics of rs2479106 genotypes(n(%)).

genotypes	AA	AG+GG	t-value	*P*-value
*n*	93	58		
Age (years)	27.014 ± 4.527	25.963 ± 4.903	1.006	0.317
BMI(kg/m^2^)	23.463 ± 3.813	23.546 ± 3.978	-0.127	0.899
FSH (IU/L)	5.976 ± 1.480	5.487 ± 1.342	1.945	0.056
LH (IU/L)	12.298 ± 5.093	14.175 ± 14.843	-1.120	0.264
LH/FSH	2.159 ± 0.952	2.335 ± 0.928	-1.115	0.267
T(ng/dL)	65.516 ± 21.509	69.229 ± 21.224	-1.037	0.301
Fasting glucose (mmol/L)	4.955 ± 0.737	4.748 ± 0.727	1.686	0.094
2h- glucose (mmol/L)	7.047 ± 4.375	6.130 ± 2.077	1.492	0.138
Fasting insulin (mIU/L)	16.007 ± 12.235	17.497 ± 18.409	-0.596	0.552
2h- insulin (mIU/L)	88.433 ± 82.361	74.258 ± 61.815	1.111	0.268
HOMA-IR	3.627 ± 2.937	3.211 ± 2.640	0.866	0.388
TC (mmol/L)	4.355 ± 0.956	4.405 ± 1.064	-0.300	0.764
TG (mmol/L)	1.449 ± 0.760	1.445 ± 0.708	0.032	0.975
HDL (mmol/L)	1.308 ± 0.428	1.335 ± 0.409	-0.377	0.707
LDL (mmol/L)	2.612 ± 2.070	2.523 ± 0.846	0.301	0.764

Abbreviations: BMI, body mass index; FSH, follicle-stimulating hormone; LH, luteinizing hormone; T, testosterone; HOMA-IR, the homeostasis model assessment of insulin resistance; TC, total cholesterol; TG, triglycerides; LDL, low-density lipoprotein; HDL, high density lipoprotein.

## Discussion

It is reported that PCOS women of different ethnicity present with different clinical manifestations [22–24]. A previous study showed that the GWAS had identified three loci, which confer risk for PCOS in Han ethnic women. The Hui ethnic group is one of large groups in China. The living and prohibition of inter-ethnic marriages suggest the distinct genetic background of Hui ethnic women. Some studies indicated that there were some genetic differences between Ningxia Hui and other ethnic population [25–26]. However, most studies about PCOS are based on Han ethnic women, more studies from women of other ethnic backgrounds and geographical origins are required. Replication is necessary to determine whether the same variants conferring risk for PCOS in Hui ethnic women.

This is the first study to report the frequencies of genotypes and alleles in three different SNPs (rs2479106, 13405728 and 13429458), which were identified by GWAS, between PCOS and healthy Hui ethnic people. And we hypothesized that SNPs were associated with PCOS in particular ethnics and genetic variation may play a role in the pathogenesis of PCOS. Our main finding was that the SNP rs13425728 in the PCOS cases were higher than control group and the levels of T, TG and LDL were statistical different between rs13425728 genotypes.

The frequencies of rs13405728 (LHCGR) genotypes and alleles were significantly different between women with PCOS and controls in Ningxia Hui ethnic women, similar with the previous GWAS study in Han Chinese people [13]. It suggested that rs13405728 might be associated with PCOS in Ningxia Hui people. Bassiouny et al [27] have found that the G935A polymorphism of LHCGR gene is associated with PCOS in an Egyptian population, which is similar to our findings. Our current findings do differ from other results in different ethnic groups. Welt et al have found that 9q33.3(rs10986105, rs10818854) was associated with PCOS in Europe population; yet, 2p16.3 (rs13405728), 2p21 (rs12468394, rs12478601, and rs13429458) and 9q33.3 (rs2479106) were not associated with PCOS [13]. Goodarzi et al confirmed that DENND1A and THADA susceptibility genes were associated with PCOS in the American population, but did not found any association between LHCGR and PCOS [14]. The different results from all over the world may be caused by the different ethnicities with different genetic background, and the same SNP loci with different genotype frequencies. Using different diagnostic criteria, and sample size may contribute to the different findings. Therefore, determinations of PCOS genetic susceptibility genes still need a large sample size, uniform diagnostic criteria, multi-ethnic areas, and more extensive and in-depth research.

The results also showed that the frequencies of genotypes and alleles in rs13429458 and rs2479106 were not statistically significant between the PCOS and control groups. These results were different from other research conducted in other regions and ethnic populations [14,16–17]. The different genotype distributions might reflect differences in genetic background, and therefore gene variants might be associated with different relative risks in different populations. Although a previous study demonstrated SNP rs13429458 and rs2479106 were associated with clinical and metabolic characteristics of in Han Chinese women [28], the current study had showed that rs13429458 and rs2479106 polymorphisms of the THADA and DENND1A genes were not involved in the pathogenesis or the phenotype of PCOS in Hui Chinese women. This indicates that there is different genetic background between THADA, DENND1A and PCOS.

It is also the first study to show the clinical and biochemical characteristics of Hui ethnic women. Interestingly, analysis revealed that the rs13405728 TT genotype in the LHCGR gene was related to the increased levels of T. As we known, hyperandrogenemia is considered one of the most important pathophysiological features in PCOS [29], and obesity aggravates menstrual irregularity and increase serum total testosterone level [30]. Androgen excess can affect the follicle growth and metabolic process, playing a key role in the occurrence and development of PCOS [31–32]. *LHCGR* encodes luteinizing hormone/choriogonadotropin receptor, which is the receptor for two glycoprotein hormones, luteinizing hormone (LH) and human chorionic gonadotropin (hCG). LH stimulates ovarian theca cells to produce testosterone. Chen RM et al [33] found the c.1703C>T mutant LHCGR leading to elevated testosterone synthesis in testicular Leydig cells. Comim et al [34] found the expression of both LHCGR and CYP17A1 protein in PCOS women was increased compared with normal ovaries, which emphasizing the importance of both factors in the etiology of androgen excess in PCOS. Whether the LHCGR gene plays an important role in the occurrence of PCOS by regulating the secretion of testosterone need to be confirmed, which is also the forthcoming work of our team.

Hyperlipidemia is one of the most common metabolic disorders in PCOS women, and about 70% of them showed dyslipidemia as triglycerides, total cholesterol, LDL increased and HDL decreased [35–36]. In this study, we found the PCOS cases with TT genotypes had a higher level of TG and LDL than those with the CC and CT genotype. Dyslipidemia is not only one of the important features of the metabolic syndrome, but also an independent risk factor for cardiovascular disease [37]. It may suggest that people carrying TT genotype of rs13405728 may to at a higher risk of hyperlipemia and additional complications.

Our previous study showed the three SNPs conferred risk for PCOS in Han ethnic women, however more studies from other ethnic backgrounds are required. It is the first report on the same variants conferring risk for PCOS in Hui ethnic women. Our study may be limited by sample size, but it could still provide useful information due to the race and geographical differences.

In conclusion, The SNP rs13425728 of LHCGR gene was associated with PCOS among Ningxia Hui women. The SNP rs13425728 was related with the regulation of T, TG and LDL, which suggests that genetic variations may have a common role in the pathogenesis of PCOS and contribute to the phenotypic character of PCOS. To further reveal susceptibility genes in the pathogenesis of women with PCOS, more studies with large sample size are required.
